# Structure of the C-terminal Region of the Frizzled Receptor 1 in Detergent Micelles

**DOI:** 10.3390/molecules18078579

**Published:** 2013-07-22

**Authors:** Shovanlal Gayen, Qingxin Li, Young Mee Kim, CongBao Kang

**Affiliations:** 1 Experimental Therapeutics Center, the Agency for Science, Technology and Research, Singapore 138669, Singapore; 2 Institute of Chemical & Engineering Sciences, Agency for Science, Technology and Research (A*STAR), Singapore 627833, Singapore

**Keywords:** helix 8, Frizzled receptor, detergent micelles, NMR

## Abstract

The C-terminal domains of the Frizzleds (FZDs) contain a short conserved motif (KTXXXW). It has been demonstrated that FZDs interacted with the PDZ domain of the cytoplasmic proteins such as Dishevelled through this motif and mutations in this motif disrupted Wnt/β-catenin signaling. We carried out structural studies for a peptide derived from the C-terminal domain of the FZD_1_ in different solvents using circular dichroism and solution NMR spectroscopy. Our results showed that this domain was unstructured in an aqueous solution and formed a helical structure in detergent micelles. Fluorescence studies suggested that the tryptophan residue (W630) in the motif interacted with micelles. The solution structure of the peptide in sodium dodecyl sulfate micelles was determined and an amphipathic helix was identified. This helix may have similar function to the helix 8 of other G protein-coupled receptors.

## 1. Introduction

G protein-coupled receptors (GPCRs) are a class of membrane proteins mediating important cellular signal transductions across the cell membrane. Structural studies for GPCRs have been conducted using X-ray crystallography and NMR spectroscopy [1,2,3]. GPCRs have a structure fold containing an N-terminal domain, a seven-transmembrane core domain and a C-terminal domain. Although the membrane topology of the transmembrane domains of GPCRs is similar, the N- and C-terminal regions of different GPCRs have various length and structures. GPCRs comprise a protein superfamily consisting of Class A, B, C and Class Frizzled receptors (FZDs).The FZDs contain ten members and smoothened receptor (SMO) [4]. The extracellular N-terminal domains of FZDs have a cystein-rich domain (CRD) followed by a hydrophobic linker with different lengths [5]. The CRD binds Wnt molecules that are important in embryonic development and stem cell maintenance [5].

**Figure 1:**
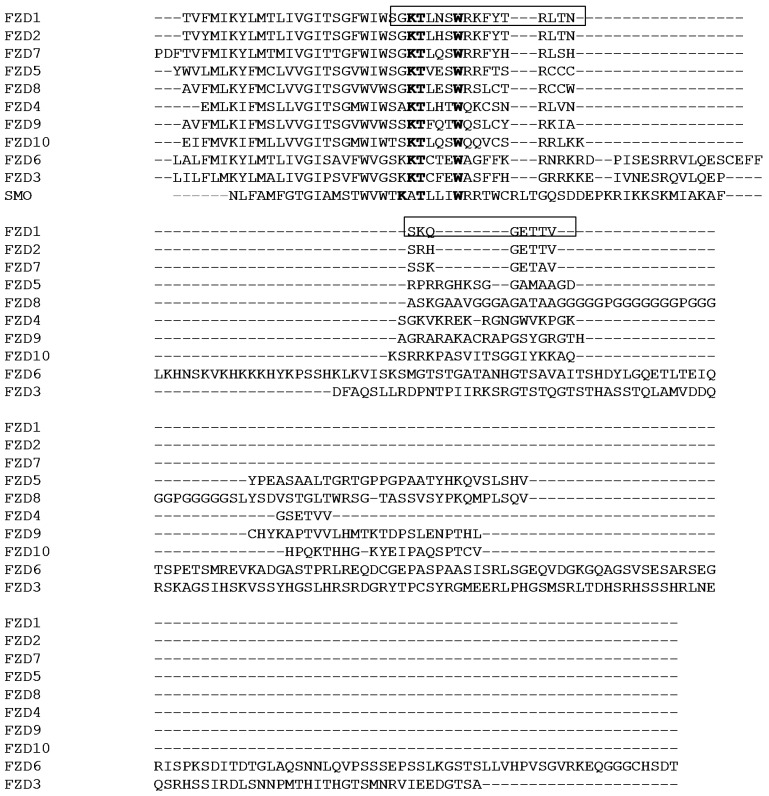
Sequence alignment of the C-terminal domains of the FZDs and SMO. The C-terminal domains from ten FZDs (FZD_1–10_) and SMO were aligned using ClustalW (http://www.ebi.ac.uk/Tools/msa/clustalw2/). The conserved KTXXXW motif is highlighted in bold. The sequence used in the study is shown with a box. The protein sequences were obtained from protein knowledgebase (http://www.uniprot.org) with the following accession numbers: FZD_1_, Q9UP38; FZD_2_, Q14332; FZD_3_, Q9NPG1; FZD_4_, Q9ULV1; FZD_5_, Q13467; FZD_6_, O60353; FZD_7_, O75084; FZD_8_, Q9H461; FZD_9_, O00144; FZD_10_, Q9ULW2; SMO, Q99835. For clarity, only a partial sequence from the C-terminus of SMO is shown.

The C-terminal regions of GPCRs interact with other proteins or help folding of the GPCRs. For class A rhodopsin-like family of GPCRs, the C-terminal region following the seventh transmembrane segment contains a helix which is amphipathic and referred as helix 8 [6]. The helix 8 normally starts after a conserved NPXXY motif in the seventh transmembrane helix. Structures of the helix 8 from different GPCRs have been studied using NMR spectroscopy [7,8,9]. In aqueous solutions, the peptide lacks secondary structures. In the presence of a membrane-mimicking environment such as detergent micelles, the peptide derived from the helix 8 forms a helical structure with hydrophobic residues interacting with detergent micelles [7,10]. The conformational changes in the presence and absence of membrane-mimicking environment may represent the activation of the receptor upon ligand binding [7,11]. The C-terminal domain following the transmembrane region of the FZDs have different length and sequence similarity (Figure 1). The domain has been shown to be necessary for localization of the receptors [12]. Studies have also demonstrated that there is a short conserved motif (KTXXXW) present in the C-terminal domain [13]. Mutations in this motif disrupted Wnt/β-catenin signaling [13]. Further study has demonstrated that this motif interacted with the PDZ domain of the cytoplasmic protein-Dishevelled (Dvl) with a low binding affinity [14]. By using a peptide scanning method, two motifs of the third intracellular loop and the C-terminal region of the FZD are identified to be important for forming a stable FDZ-Dvl complex [15].

Due to the importance of the C-terminal domain of the FZDs in the Wnt/β-catenin signaling, it will be useful to understand their structures. Compared with class A rhodopsin-like family of GPCRs, there is no NPXXY motif present in the seventh transmembrane helix of the FZDs (Figure 1). If the C-terminal domain contains a helix 8 is still unknown. In this study, we conducted structural analysis of the C-terminal region of the FZD_1_ consisting of 25 residues (Figure 1). Our results indicated that the peptide was not structured in an aqueous solution, but formed a helical structure in detergent micelles such as sodium dodecyl sulfate (SDS) and dodecylphosphocholine (DPC). Sequence analysis and structural studies indicated that the C-terminal domain of FZD_1_ contains an amphipathic helix which is similar to other GPCRs. Residue such as Trp in this domain is interacting with micelles. Our result will be useful to understand the role of the C-terminal domains of FZDs in signal transductions.

## 2. Results and Discussion

The FZDs are unconventional GPCRs and classified as a novel family of GPCRs [4]. For the conventional GPCRs, the C-terminal domain is important for signaling and G protein coupling. Examples include rhodopsin and CB1 receptors [16,17]. The C-terminal domains of FDZs were also shown to involve in signal transduction, while its structure is still unknown.

### 2.1. Selection of the C-terminal Domain of FZD_1_

The C-terminal domains from the FZDs have different length and sequence diversity (Figure 1). To understand the structure, we focused on the FZD_1_ because it has a relatively short sequence which is suitable for NMR studies. Peptide corresponding to S623 to V647 was synthesized and purified (Figure 1). Further studies were carried out to understand its structure under different conditions.

### 2.2. Circular Dichroism (CD) Experiment

**Figure 2:**
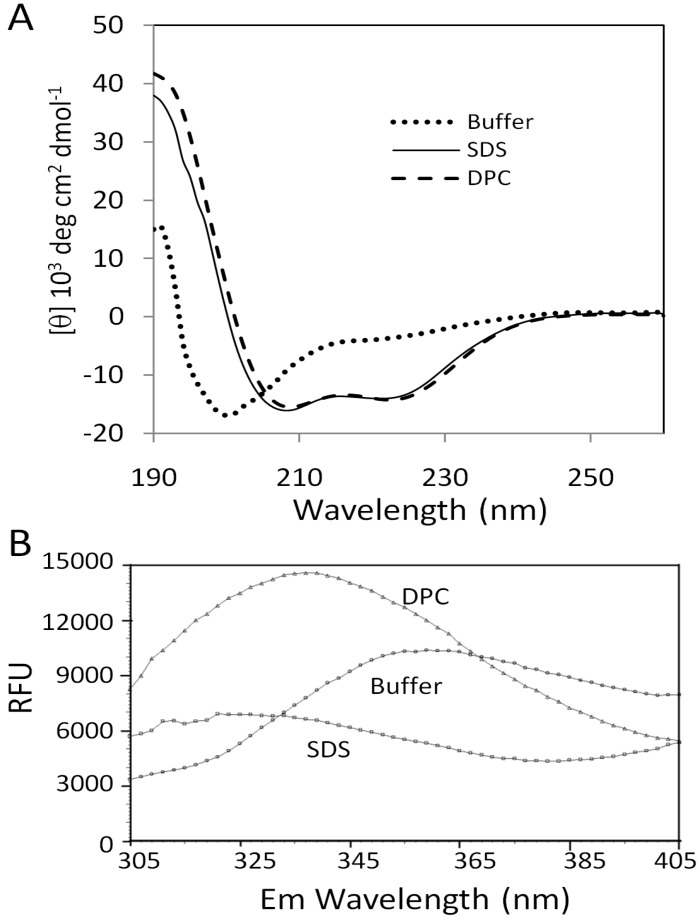
Effect of solvent systems on the peptide structure. (**A**) CD spectra of the peptide in different solutions. The CD spectra of the peptide in an aqueous solution, DPC and SDS micelles are shown with dotted, dashed and solid lines, respectively. (**B**) Fluorescence spectroscopy for the peptide. The emission spectra for peptide in different solutions are shown.

Far-UV CD was first employed to examine the conformations of peptide derived from FZD_1_ under different conditions. CD result showed that when the peptide was prepared in an aqueous solution containing only 20 mM sodium phosphate, pH7.0, the peptide was unstructured (Figure 2A). In the presence of detergent micelles such as SDS and DPC that were used as membrane mimetics [18], the CD spectra of the peptide in these two detergent micelles changed obviously with spectra characterized for an α-helix in which double minima at 222 and 208 nm were observed (Figure 2A). Further data analysis using the K2D2 server demonstrated that peptide acquired similar helical conformation in these two detergent solutions. It was predicted from the K2D2 server that the helical content was approximately 70% in these two solutions. Micelle-dependent conformational change has been demonstrated for several peptides derived from GPCRs and other membrane proteins [7,8,19]. Our result suggested that the C-terminal region of the FZD_1_ could form an α-helix in the presence of detergent micelles mimicking conditions of cell membrane proximity.

### 2.3. Fluorescence Analysis

To understand the interaction between the peptide and detergent micelles, fluorescence spectroscopy was carried out. It is known that the fluorescence emission spectrum of tryptophan residues is very sensitive to the environment, which can be used to measure residue and membrane interactions. In an aqueous solution, the emission maximum is close to 350 nm after excitation at 280 nm. If the tryptophan residue is buried in a hydrophobic environment such as in the presence of micelles, the emission maximum will be shifted to a lower wavelength [20]. The peptide in the aqueous solution had an emission maximum of 350 nm, indicating that the W630 was exposed to the solvent (Figure 2B). In the presence of the DPC micelles, the emission maximum shifted to ~330 nm, indicating that the residue W630 was buried in the DPC micelles. Although there was no clear emission maximum observed when SDS micelles were present, the disappearance of maximum at 350 nm indicated that the tryptophan residue was buried in or affected by SDS micelles (Figure 2B).

### 2.4. NMR Study

**Figure 3:**
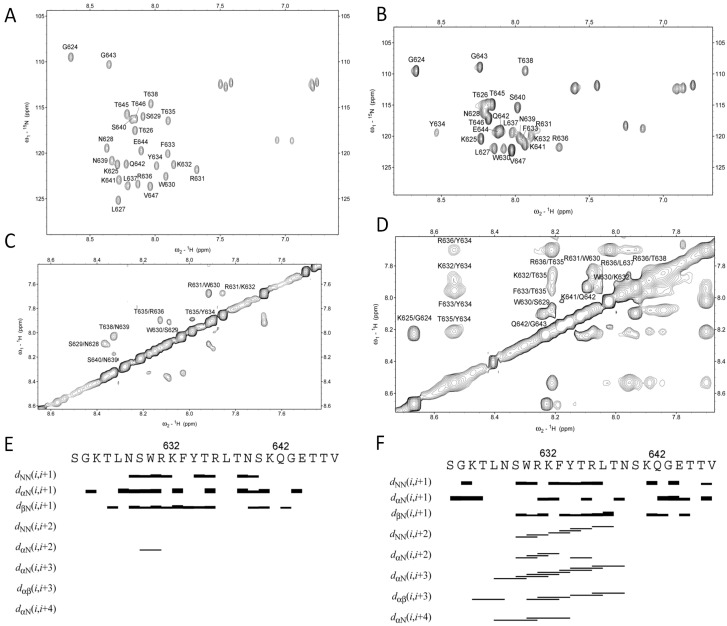
NMR analysis of the C-terminal region of the FZD_1_. Assignments for the ^15^N-HSQC spectra of the peptide in the phosphate buffer (**A**) and in SDS micelles (**B**) are shown. NOESY spectra of the peptide in the phosphate buffer (**C**) and in SDS micelles (**E**) are shown. The NOEs between different residues are labeled residues name and sequence number. The NOE connections of the peptide in the aqueous solution (**E**) and in SDS (**F**) micelles are shown.

SDS or DPC were used as a membrane mimetic in membrane protein structural studies [18,21]. To study the structures of the peptide in the presence and absence of detergent micelles, NMR data for peptide dissolved in different solutions were collected. The peptide produced poorly dispersed spectra in DPC micelles which may due to the size of the peptide- DPC micelles (data not shown). Resonance assignments for the peptide in the aqueous solution and SDS micelles were obtained (Figure 3). Although peptide in aqueous solution exhibited dispersed cross-peaks in a ^15^N-HSQC spectrum, further NOE connection indicated that this peptide was not structured because only *d*_NN_ (*i*, *i*+1) connections were observed (Figure 3E). This result is consistent with the CD data. When the peptide was dissolved in SDS micelles, we conducted NMR experiments at 313 K because the signal was improved compared to 298 K. NOE connections indicated that residues L627 to N639 formed a helix, which was confirmed by the presence of αN (*i*, *i*+3) and αN (*i*, *i*+4) NOE connections (Figure 3). SDS micelles have been used as a membrane-mimicking system in structural studies of several membrane proteins [18]. The data suggested that peptide may form a helix when it is close to the cell membrane.

### 2.5. Structure Determination

**Figure 4:**
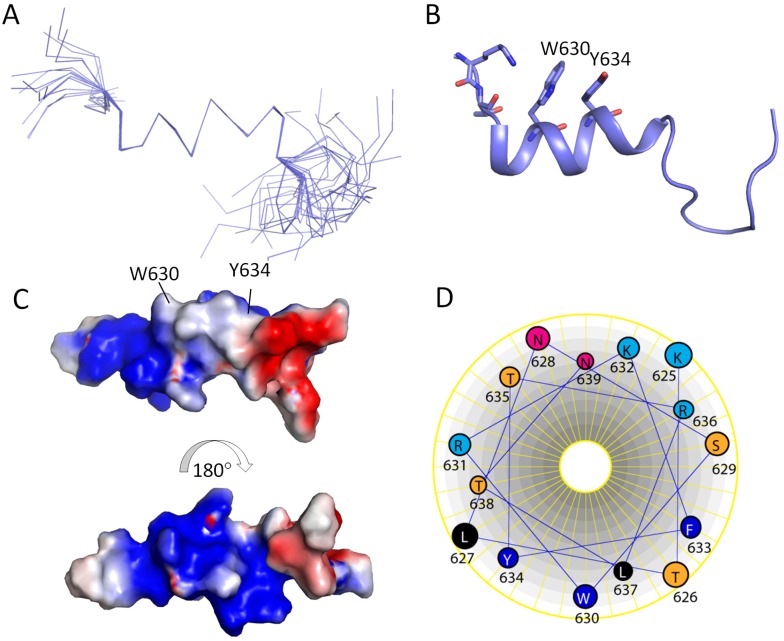
Structure of the peptide in SDS micelles. (**A**) Superimposed structures with lowest energy. Only backbone atom traces are shown. (**B**) Cartoon representation of the structure. The Trp and Tyr residues are shown in sticks. (**C**) Surface representation of the peptide structure. The charge representation of the helical surface and the figures were made using PyMOL (http://www.pymol.org). (**D**) Helix wheel representation for the helix.

The structure of the peptide in SDS micelles was determined based on 104 NOEs (Table 1). Figure 4 shows the 20 superimposed structures with lowest energy. The root-mean-square deviation (RMSD) for the backbone atoms from the helix of the twenty structures was 0.4 Å (Table 1). The restraints used in this study and the structural analysis using PROCHECK-NMR [22] are shown in Table 1. Residues L627 to N639 formed a helix and the C-terminus of this peptide was not structured (Figure 4). Helix wheel representation for this helix indicated that it behaves like an amphipathic helix (Figure 4). The hydrophobic residues such as W, L and Y are facing one side and charged residues such as R and N are facing the other side (Figure 4). These hydrophobic residues may be important for interacting micelles, for example, the Trp residue was buried in the micelles from our fluorescence experiment (Figure 2B).

Solvent-induced conformational changes have been observed in C-terminal domains of other GPCRs such as human β2 adrenergic receptor [8] and CB1 cannabinoid receptor [7]. In those receptors, their C-terminal domains contain a helix 8 following the seventh transmembrane helix. Helix 8 in GPCRs may serve as a membrane anchor because the helix is amphipathic and contains palmitoylation sites that can stabilize the interaction with membrane. The conformation of the helix may be regulated by allosteric modulators binding to the seven-transmembrane core domain. After activation by an allosteric ligand, the helix 8 may become exposed to form a disordered structure to expose its binding site for other proteins [7]. In GPCRs such as rhodopsin, the hydrophobic residues in this motif may stack against each other to keep the receptor in a pre-receptive state [6,23]. Unlike other GPCRs, there is no NPXXY motif present in the seventh transmembrane helix of FZDsFrom our studies, it was demonstrated that the C-terminal domain of FZD_1_ had similar characteristics to the helix 8 from other GPCRs such as CB1 receptor.

**Table 1:** Experimental distance and statistics for the 20 structures determined in SDS micelles.

Restraints	
Intraresidue NOEs	71
Sequential NOEs (i to i+1)	65
Medium range NOEs (i to i + 2,3,4)	39
Dihedral angle restraints	0
Number of NOE violations >0.5 Å	0
Ramachandran plot (%)	
Residues in most favored regions	55.8
Residues in additional allowed regions	38.8
Residues in generously allowed regions	5.7
Residues in disallowed regions	0
Structural statistics	
RMSD for backbone atoms (6-17)	0.4 Å
RMSD for heavy atoms (6-17)	0.86 Å

**Figure 5:**
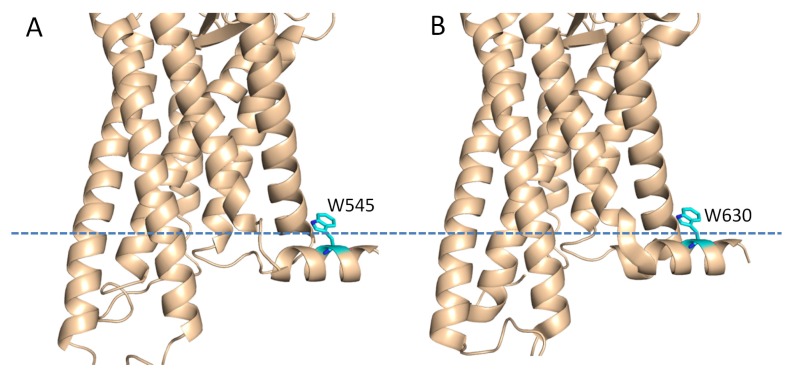
Role of the Trp residues in membrane binding. A. Crystal structure of the SMO receptor. B. Homology model of the FZD_1_ using the crystal structure of the SMO receptor as a template [25]. The possible membrane interface is shown in dash line. The residues in both structures are shown in sticks.

The structure of the smoothened (SMO) receptor which shows sequence similarity to the FZDs was determined using X-ray crystallography. SMO also has a conserved KATXXXW motif at the C-terminus [24] and this motif has been described to stabilize the receptor by packing parallel to the membrane (Figure 5). The tryptophan residue (W545) localized at the membrane interface, which is similar to what we observed in the C-terminal domain of the FZD_1_. The C-terminal domain of FZDs contains a conserved KTXXXW motif lacking an alanine residue for PDZ domain interactions [13] (Figure 1). From our study, this motif had a tendency to form a helix. Although the KT residues within this motif are not helical under current conditions, this may arise from the fact that this peptide did not include the seventh transmembrane helix. The tryptophan residue (W630) is involved in the micelle interactions, which was confirmed from the fluorescence study (Figure 2B). Mutations in this motif may disrupt the helical structure or affect membrane interaction [13], which in turn will affect the signal transduction or binding to Dvl [15]. All these results suggest that the C-terminal domains of FZDs might have similar role to those of the conventional GPCRs. There is a cystein residue present in the helix 8 of GPCRs. This cystein can be modified by palmitoylation, which could serve as a lipid anchor. As there is no cystein present in the C-terminus of FZDs, this tryptophan residue may act as a lipid anchor to stabilize the interaction with membrane. Under different conditions, this domain interacts with Dvl or membrane, which will produce different signals for the downstream pathways. Previous studies have proposed that the helix 8 in GPCRs is involved in G-protein coupling and GPCR activation [10]. The sequence similarity within FZDs suggested that the helix 8 may be a common structural feature within this family (Figure 1). This region might have the similar functional roles to the helix 8 of the classical GPCRs. Our study provided structural basis to understand its function.

## 3. Experimental

### 3.1. Sample Preparation

The peptide sequence was SGKTLNSWRKFYTRLTNSKQGETTV and was derived from the C-terminal domain of the FZD_1_ corresponding to residue 623 to 647. This peptide was synthesized and purified from GL Biochem Ltd (Shanghai, China) with more than 95% purity. The peptide was prepared to a concentration of 5 mg/mL in a buffer containing 20 mM sodium phosphate, pH 7.0 or same buffer that contained 2% deuterated DPC or SDS. The samples with 10% D_2_O were transferred into 3-mm NMR tubes for data acquisition. 

### 3.2. Circular Dichroism (CD) Spectroscopy

For secondary structural analysis, samples were diluted to 0.2 mg of peptide per ml for CD analysis as described previously [19]. Peptide was dissolved in sodium phosphate buffer in the present and absence of detergent micelles. All the CD experiments were conducted using a Chirascan™ Circular Dichroism Spectrometer at 25 °C. The CD spectra were recorded in a continuous mode with a 1-nm data pitch. The secondary structure was analyzed using the K2D2 server (http://www.ogic.ca/projects/k2d2/).

### 3.3. Fluorescence Spectroscopy

The effect of the solvent on the trytophan residue was measured using the similar method described [20]. The fluorescence emission spectra were measured in a 96-well plate. Peptide was dissolved in the buffer to a concentration of 0.1 mg/mL. Samples with 100 μL volume in the presence and absence of micelles were subjected to analysis. Excitation wavelength was 280 nm and the emission was scanned from 305 to 405 nm. Experiment was carried out at 37 °C.

### 3.4. NMR Experiments

The NMR data were collected on a Bruker Avance II spectrometer with a proton frequency of 700 MHz equipped with a cryoprobe. A total correlation spectroscopy (TOCSY) experiment was recorded with a mixing time of 80 ms [26]. Two-dimensional (2D) nuclear overhauser effect spectroscopy (NOESY) experiments were recorded with mixing times of 100 ms, 200 ms and 300 ms, respectively. The experiments for peptide in sodium phosphate buffer were performed at 298 K. In the presence of micelles, the TOCSY and NOESY spectra were recorded at 313 K to increase the resolution. Proton chemical shift values were referenced directly to 4, 4-dimethyl-4-silapentane-1-sulfonic acid (DSS). All of the spectra were processed using Topspin 2.1 and analyzed using Sparky (http://www.cgl.ucsf.edu/home/sparky/).

### 3.5. Resonance Assignment and Structure Determination

The resonance assignment for the peptide was obtained using the procedure including the identification of spin systems in a TOCSY spectrum and spin connections in a NOESY spectrum [26,27]. The sequential connectivity of the residues was confirmed based on the connectivity in the HN-HN or Hα-HN region. A ^1^H-^15^N heteronuclear single quantum correlation spectroscopy (HSQC) in ^15^N natural abundance was acquired and processed to facilitate resonance assignment. The NOE peaks picked from a NOESY spectrum with a mixing time of 200 ms were integrated for structural determination. The peaks were assigned manually and calibrated to distances using CYANA 2.1 for structural determination [28]. Structure determination for peptide in SDS micelles was conducted as described previously [26]. The structure was calculated using torsion angle dynamics simulated annealing as implemented in CYANA using NOE restraints [26,28]. One hundred structures were calculated and twenty of the structures with lowest energies were analyzed with MOLMOL [29] and displayed using PyMOL (http://www.pymol.org).

## 4. Conclusions

In summary, we carried out structural studies for a peptide derived from the C-terminal domain FZD_1_. Our results show that the peptide adopted different conformations under different solvent conditions. In the aqueous solution, it was not structured. It formed a helical structure when a membrane-mimicking detergent was present. The tryptophan residue (W630) in the KTXXXW motif may be important for membrane interaction.
